# Supplementary material for: A neural network for modeling human concept formation, understanding and communication
Source: Nat Comput Sci. 2026 Feb 19;6(5):497–511. doi: 10.1038/s43588-026-00956-4 (PMC13216063; doi:10.1038/s43588-026-00956-4)
Supplement: Supplementary file 1 — Supplementary Figs. 1–4, and ablation study and robustness analysis of model–brain fitting. [file 43588_2026_956_MOESM1_ESM.pdf]

nature computational science

Supplementary information https://doi.org/10.1038/s43588-026-00956-4

A neural network for modeling human concept formation, understanding and communication

In the format provided by the
authors and unedited

Contents

1 Ablation studies on CATS Net architecture 3
1.1 Backbone Architecture . . . 3
1.2 Concept Space Dimensionality . . . 3
1.3 Network Depth . . . 3
1.4 Training Strategy . . . 3

2 Ablation studies on concept space construction 4
2.1 Comparison with one-hot vectors . . . 4
2.2 Learnability of the concept space and CA/TS capacity are both crucial 4

3 Robustness analysis of model-brain correlations 5
3.1 Model initialization . . . 5
3.2 Datasets splits . . . 5
3.3 Semantic ontologies . . . 5

List of Figures

Supplementary Figure 1: | **Ablation study on CATS Net. a,** Ablation studies and hyperparameter explorations on backbone, concept size, number layers of CA/TS module and training strategy. The left most 2 bars was adopted from Figure 2a, while the others represents the average of mean accuracy across 5 independently initialized models after training, and each point represents the corresponding mean accuracy cross all categories (from the 3rd bar to the right most one: using ViT as backbone, setting concept size to 10, setting concept size to 100, setting the layer number of CA/TS module to 1, setting the layer number of CA/TS module to 5, end-to-end training of concept vectors together with CA/TS module). **b,** Ablation studies on concept space construction. The left most one bar was adopted from Figure 2a, while the others represents the average of mean accuracy across 5 independently initialized models after training, and each point represents the corresponding mean accuracy cross all categories (from the 2nd bar to the right most one: setting concept size to 100, setting concept size to 1000 using fixed one-hot vectors, setting concept to fixed Word2Vec vectors projected to 20 dimensions, setting concept to fixed 20-dimension random vectors, setting concept to fixed 20-dimension random vectors with 1 CA/TS layer, setting 20-dimension concept vectors to be learnable with 1 CA/TS layer). . . . 7

Supplementary Figure 2: | **Maximum correlations between CATS instances and all SPOSE49 dimensions.** Each bar represents the maximum Pearson correlation between the 20 concept dimensions of a given CATS instance and each SPOSE49 dimension (dimension labels shown around the perimeter; dimension names from Hebart et al. [1]). The red circle indicates the significance threshold ($r = 0.107$, two-tailed $p < 0.05$, df = 330). 8

Supplementary Figure 3: | **Translation Module Analyses. a,** For this translation module (”apple” category was withheld from the student Net’s training), given all 100 teacher concept vectors as input, we recorded the layer-wise activation and conducted layer-wise RDM (Pearson’s correlation). **b,** The layer-wise RDM Spearman’s correlation similarity matrix based on (a). **c,** The average layer-wise RDM Spearman’s correlation similarity across all 100 translation modules. **d,** One-sample t-test of each value at translation module group level. . . 9

Supplementary Figure 4: | **Searchlight RSA results within the VOTC mask for three common semantic categories (animals, large non-manipulable objects, small manipulable objects).** The maps show t-values reflecting the model-instance-level correspondence between the CATS concept layer representations (n=30) and brain activity of 26 subjects. Results are thresholded at voxel-level $p < 0.001$ (one-tailed) and cluster-level family-wise error (FWE) corrected $p < 0.05$. The color scale represents the t-statistic values. 10

1 Ablation studies on CATS Net architecture

1.1 Backbone Architecture

We tested our framework with different visual backbones beyond ResNet50, including Vision Transformer (ViT-B/16). The results demonstrate that CATS Net maintains consistent performance across different feature extractors, achieving similar accuracy (0.966, the third bar from the left in Supplementary Figure 1a) regardless of the backbone choice. This indicates that our hierarchical gating mechanism is robust and not dependent on specific CNN architectures.

1.2 Concept Space Dimensionality

First, our selection of a 20-dim concept space is grounded in established neuroscientific literature on human conceptual representations. As we described in our manuscript, *First is concept formation: The higher-dimensional sensory-motor experience is compressed into lower-dimensional representational spaces [2–5], whose dimensionality typically ranges from 20 to several hundreds [6–10].* This neurobiological evidence suggests that human concept formation involves compressing higher-dimensional sensory-motor experience into lower-dimensional representations, with 20 dimensions falling well within the empirically observed range for effective conceptual encoding in biological systems. Our choice thus aligns with the natural dimensionality constraints observed in human conceptual processing.

Second, we empirically validated this architectural choice through systematic ablation studies. We conducted experiments across different concept space dimensions (10, 20, and 100 dimensions) and found that performance remained remarkably consistent, with 20-dimension providing an optimal balance between representational efficiency and compression capability (the second, fourth and fifth bar from the left in Supplementary Figure 1a). The convergence between neurobiological evidence and our empirical findings strengthens the theoretical foundation of our approach.

1.3 Network Depth

We tested CA/TS modules with 1, 3, and 5 layers. The results demonstrate that our framework is remarkably robust to depth variations, with all configurations achieving comparable performance (the sixth and seventh bar from the left in Supplementary Figure 1a).

1.4 Training Strategy

We compared our alternating two-phase training with end-to-end joint training. Interestingly, both approaches yielded nearly identical results, suggesting that the concept formation process is robust to training methodology (the last bar from the left in Supplementary Figure 1a). The learning of concept space can be independent from the learning of network parameters. We believe this approach aids readers in understanding that once CA/TS has been trained, new functional and meaningful network

configurations can be directly obtained by acquiring concept vectors solely in the concept space. As demonstrated in our leave-one-out experiments and communication experiments, this process does not involve modifications to network parameters, but only involves operations in the concept space.

These comprehensive ablation studies demonstrate that CATS Net's robustness, which indicates that our framework captures a general computational principle for concept formation that is broadly applicable across different implementation details.

2 Ablation studies on concept space construction

We compared the performance of different types of concept spaces on the ImageNet-1k binary judgment task. The results are as follows:

2.1 Comparison with one-hot vectors

First, increasing dimensions through one-hot is not optimal from a computational performance perspective. The results in Supplementary Figure 1b demonstrated that a learnable 100-dim concept (the second bar from the left) can achieve better results (mean difference = 0.0043, 95% bootstrap CI [0.0021, 0.0056], 5000 resamples, two-sided permutation test with 10,000 permutations, p = 0.0079) than a 1000-dim one-hot (the third bar from the left). Second, one-hot has poor scalability. One-hot vectors are orthogonal, which means its dimension scales linearly with the number of classes. This is inefficient and biologically implausible for a large number of concepts. Therefore, in summary, whether from the perspective of overall performance or scalability, one-hot vectors are not a better choice.

2.2 Learnability of the concept space and CA/TS capacity are both crucial

Trainable 20-dim concept space outperforms the frozen 20-dim random vectors (mean difference = 0.0192, 95% bootstrap CI [0.0185, 0.0200] with 5000 resamples, two-sided permutation test with 10,000 permutations, p ¡ 0.001) and the frozen 20-dim Word2Vec vectors (mean difference = 0.0279, 95% bootstrap CI [0.0256, 0.0313] with 5000 resamples, two-sided permutation test with 10,000 permutations, $p < 0.001$). Indeed, given the frozen 20-dim random vectors, the CA/TS modules are re-organizing its weights to accommodate a fixed arbitrary space. When we reduced the number of CA/TS layers from 3 to 1, we can clearly see that the accuracy drops from 0.944 (the fourth bar from the left in Supplementary Figure 1b) to 0.793 (the fifth bar from the left in Supplementary Figure 1b), indicating that the current CA/TS with limited capacity is not sufficient to accurately complete the task in the fixed random concept space. However, if we permit concept vector learning in this setting (i.e., with one layer of the CA/TS module), the accuracy rises back to 0.954 (the last bar from the left in Supplementary Figure 1b), which is close to the case of using 3 layers of CA/TS for 20-dim concept vectors (the first bar from the left in Supplementary Figure 1b). This shows that in essence, the CATS Net requires the learnability of the concept space

and the capacity of CA/TS to support it. When the CA/TS capacity is limited, the learnability of the concept space becomes crucial.

3 Robustness analysis of model-brain correlations

3.1 Model initialization

We independently trained 30 models and obtained highly consistent correlation results at the group level using Fisher-z transformed, ceiling-corrected correlations: concept–VOTC, $t(29) = 9.27$, $p < 0.001$, Cohen's $d = 1.70$; CA1–Semantic Control, $t(29) = 6.44$, $p < 0.001$, Cohen's $d = 1.18$).

3.2 Datasets splits

Additionally, we computed the instance-average model-brain correspondence per participant (averaging across the 30 independently initialized models). In VOTC, correlation values between the concept layer of all 30 models and each subject were greater than zero, with an overall average of mean $\rho\pm$ SE $= 0.095 \pm 0.009$. At the group statistical level, this effect was significant ($t(25) = 10.90$, $p < 0.001$, Cohen's $d = 2.14$), indicating that the model's semantic representations can stably correspond to human VOTC representations across subjects.

In the semantic control network, we similarly observed consistent positive correlations between the CA1 layer of 30 models and subjects' neural representations ($t(25) = 3.51$, $p < 0.001$, Cohen's $d = 0.69$). This further supports the correspondence between the control module in the CATS framework and the semantic control network. In addition, the CA1 layer of all thirty models also demonstrated significant fitting advantages for the Semantic Control Network in each subject, relative to the Multiple Demand Network ($t(25) = 2.23$, $p = 0.035$, Cohen's $d = 0.43$).

3.3 Semantic ontologies

Since WT95 contains three classic human semantic categories (animals, large non-manipulable objects, and small manipulable objects), we divided it into three subsets and conducted analyses within each subset following the same RSA-searchlight analysis procedure. Specifically, we generated neural RDMs based on stimuli within each subset and compared them with feature RDMs from the model's concept layer. Given that previous whole-brain analyses already demonstrated high correspondence between the concept layer and human VOTC, this analysis was confined within a predefined VOTC mask. Results showed that in each semantic category, CATS models captured significant neural effects within VOTC and demonstrated unique advantages relative to sensory input layer representations (ResNet-50 output) (voxel-level $p < 0.001$, one-tailed; cluster-level FWE-corrected $p < 0.05$; see Supplementary Figure 4).

Specifically, for the animal category, our analysis identified significant clusters in bilateral fusiform gyrus (FG), extending to the lateral occipital complex (LOC). For large non-manipulable objects, we detected three spatially relatively independent significant clusters: one located in the left occipital pole (OP), another in the right occipital pole extending anteriorly to the lingual gyrus (LING) and medial FG, and

a third in the left medial FG. Finally, for small manipulable objects, we identified a significant cluster located in the left FG. The weak effect may come from dataset composition—few representative tool exemplars and marked within-category heterogeneity (spanning small, rounded objects, e.g., buttons, to elongated objects, e.g., fishing rods).

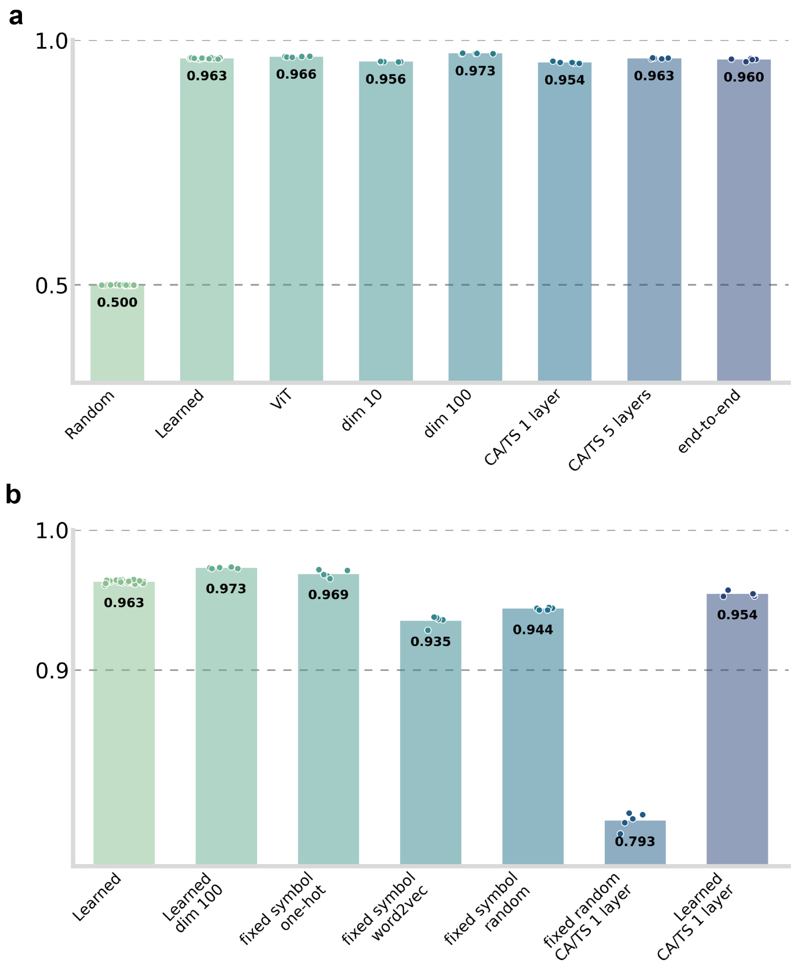

Supplementary Figure 1. | **Ablation study on CATS Net. a,** Ablation studies and hyperparameter explorations on backbone, concept size, number layers of CA/TS module and training strategy. The left most 2 bars was adopted from Figure 2a, while the others represents the average of mean accuracy across 5 independently initialized models after training, and each point represents the corresponding mean accuracy cross all categories (from the 3rd bar to the right most one: using ViT as backbone, setting concept size to 10, setting concept size to 100, setting the layer number of CA/TS module to 1, setting the layer number of CA/TS module to 5, end-to-end training of concept vectors together with CA/TS module). **b,** Ablation studies on concept space construction. The left most one bar was adopted from Figure 2a, while the others represents the average of mean accuracy across 5 independently initialized models after training, and each point represents the corresponding mean accuracy cross all categories (from the 2nd bar to the right most one: setting concept size to 100, setting concept size to 1000 using fixed one-hot vectors, setting concept to fixed Word2Vec vectors projected to 20 dimensions, setting concept to fixed 20-dimension random vectors, setting concept to fixed 20-dimension random vectors with 1 CA/TS layer, setting 20-dimension concept vectors to be learnable with 1 CA/TS layer).

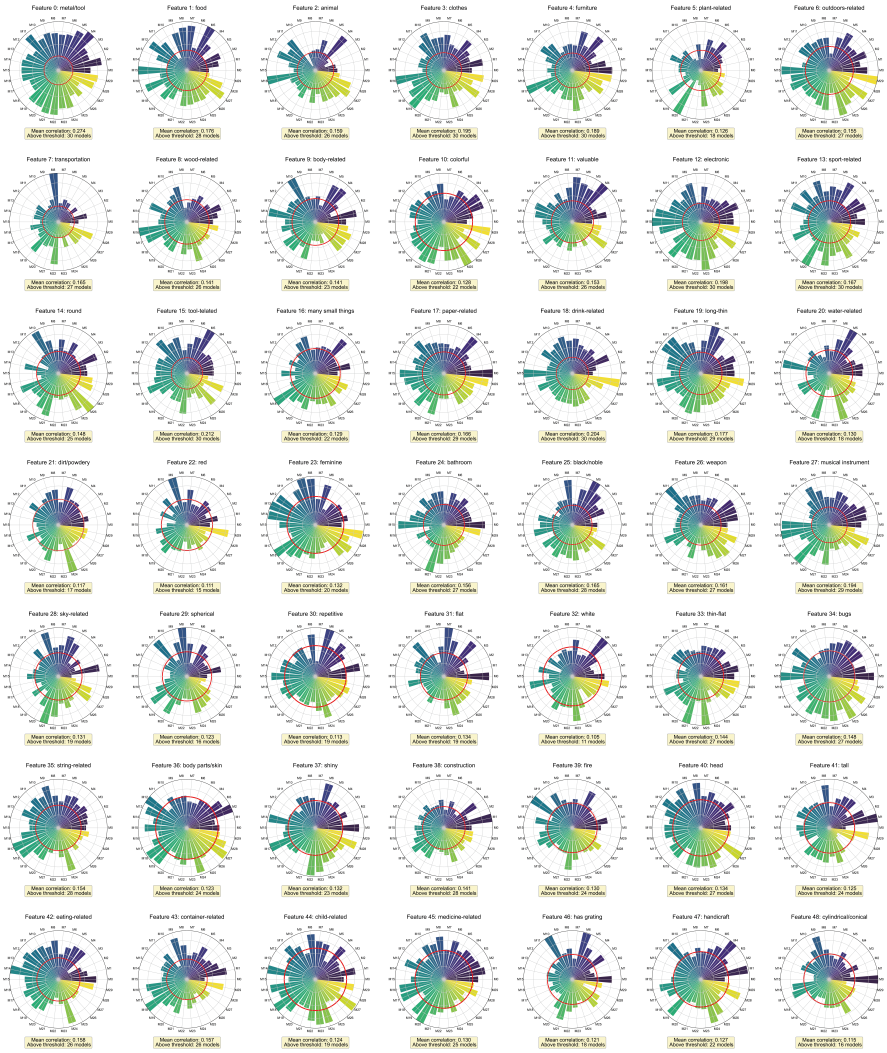

Supplementary Figure 2. | Maximum correlations between CATS instances and all SPOSE49 dimensions. Each bar represents the maximum Pearson correlation between the 20 concept dimensions of a given CATS instance and each SPOSE49 dimension (dimension labels shown around the perimeter; dimension names from Hebart et al. [1]). The red circle indicates the significance threshold ($r = 0.107$, two-tailed $p < 0.05$, df = 330).

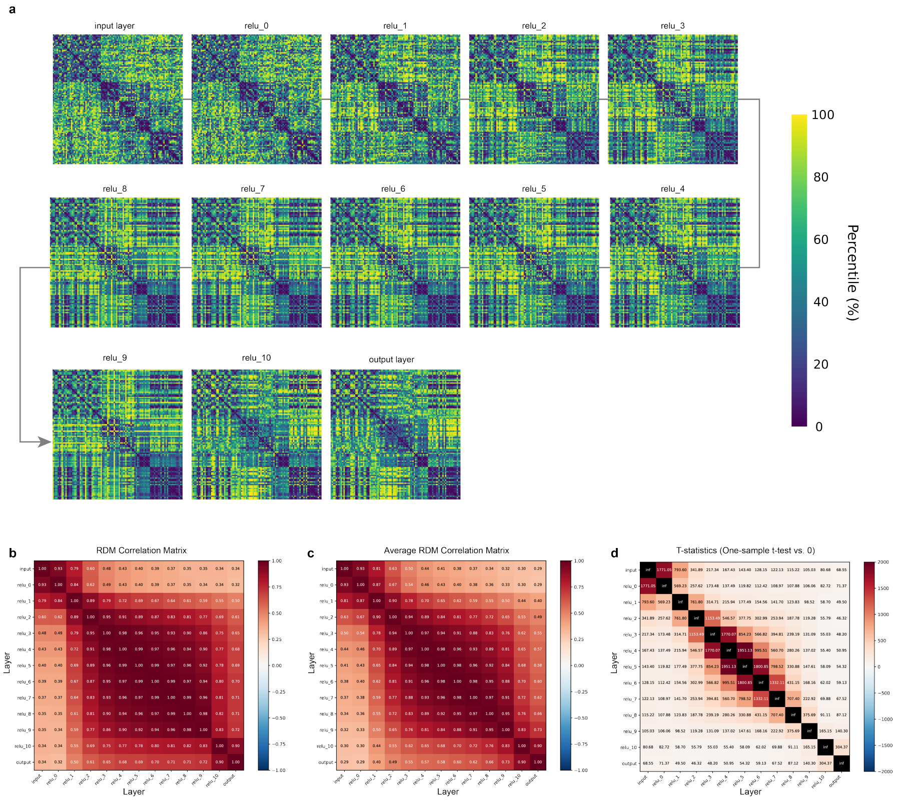

Supplementary Figure 3. | **Translation Module Analyses.** **a,** For this translation module (”apple” category was withheld from the student Net’s training), given all 100 teacher concept vectors as input, we recorded the layer-wise activation and conducted layer-wise RDM (Pearson’s correlation). **b,** The layer-wise RDM Spearman’s correlation similarity matrix based on (a). **c,** The average layer-wise RDM Spearman’s correlation similarity across all 100 translation modules. **d,** One-sample t-test of each value at translation module group level.

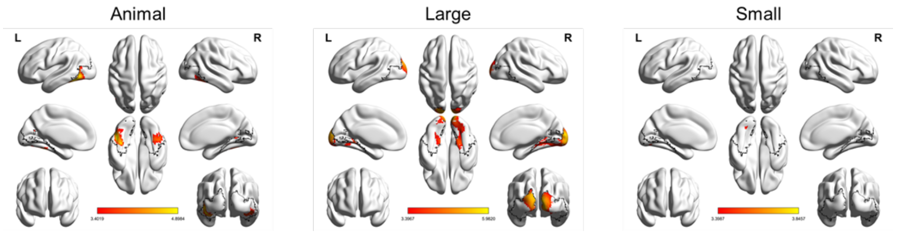

Supplementary Figure 4. | Searchlight RSA results within the VOTC mask for three common semantic categories (animals, large non-manipulable objects, small manipulable objects). The maps show t-values reflecting the model-instance-level correspondence between the CATS concept layer representations (n=30) and brain activity of 26 subjects. Results are thresholded at voxel-level $p < 0.001$ (one-tailed) and cluster-level family-wise error (FWE) corrected $p < 0.05$. The color scale represents the t-statistic values.

References

[1] Hebart, M.N., Zheng, C.Y., Pereira, F., Baker, C.I.: Revealing the multidimensional mental representations of natural objects underlying human similarity judgements. Nat Hum Behav **4**(11), 1173–1185 (2020)
