# Supplementary material for: A neural network for modeling human concept formation, understanding and communication
Source: Nat Comput Sci. 2026 Feb 19;6(5):497–511. doi: 10.1038/s43588-026-00956-4 (PMC13216063; doi:10.1038/s43588-026-00956-4)
Supplement: Supplementary file 2 — Reporting Summary [file 43588_2026_956_MOESM2_ESM.pdf]

Corresponding author(s): Shan Yu; Yanchao Bi; Yang Chen.

Last updated by author(s): Jan 3, 2026

Reporting Summary

Nature Portfolio wishes to improve the reproducibility of the work that we publish. This form provides structure for consistency and transparency in reporting. For further information on Nature Portfolio policies, see our Editorial Policies and the Editorial Policy Checklist.

Statistics

For all statistical analyses, confirm that the following items are present in the figure legend, table legend, main text, or Methods section.

n/a	Confirmed	
☐	☒	The exact sample size (n) for each experimental group/condition, given as a discrete number and unit of measurement
☐	☒	A statement on whether measurements were taken from distinct samples or whether the same sample was measured repeatedly
☐	☒	The statistical test(s) used AND whether they are one- or two-sided<br>*Only common tests should be described solely by name; describe more complex techniques in the Methods section.*
☐	☒	A description of all covariates tested
☐	☒	A description of any assumptions or corrections, such as tests of normality and adjustment for multiple comparisons
☐	☒	A full description of the statistical parameters including central tendency (e.g. means) or other basic estimates (e.g. regression coefficient) AND variation (e.g. standard deviation) or associated estimates of uncertainty (e.g. confidence intervals)
☐	☒	For null hypothesis testing, the test statistic (e.g. F, t, r) with confidence intervals, effect sizes, degrees of freedom and P value noted<br>*Give P values as exact values whenever suitable.*
☒	☐	For Bayesian analysis, information on the choice of priors and Markov chain Monte Carlo settings
☒	☐	For hierarchical and complex designs, identification of the appropriate level for tests and full reporting of outcomes
☐	☒	Estimates of effect sizes (e.g. Cohen's d, Pearson's r), indicating how they were calculated

Our web collection on statistics for biologists contains articles on many of the points above.

Software and code

Policy information about availability of computer code

Data collection	No software was used
Data analysis	Brain Analysis:<br>The functional images were preprocessed and analyzed using Statistical Parametric Mapping (SPM12; http://www.fil.ion.ucl.ac.uk/spm). After preprocessing, data were analysed using SPM12 and Python (version 3.10).<br>The analysis and deep learning model codes based on Python (PyTorch 2.01) and are available on GitHub and Zenodo (https://doi.org/10.5281/zenodo.18136642).

For manuscripts utilizing custom algorithms or software that are central to the research but not yet described in published literature, software must be made available to editors and reviewers. We strongly encourage code deposition in a community repository (e.g. GitHub). See the Nature Portfolio guidelines for submitting code & software for further information.

Data

Policy information about availability of data

All manuscripts must include a data availability statement. This statement should provide the following information, where applicable:

- Accession codes, unique identifiers, or web links for publicly available datasets
- A description of any restrictions on data availability
- For clinical datasets or third party data, please ensure that the statement adheres to our policy

Source data are provided with this paper. The fMRI data that support the findings of this study have been deposited in the Open Science Framework (OSF) at https://osf.io/5y8p6/overview. The embeddings of SPOSE49 model are available via OSF at https://osf.io/f5rn6/files/8yjh5. Additionally, the anchor word embeddings used for the Binder65 model can be accessed at https://www.neuro.mcw.edu/index.php/resources/brain-based-semantic-representations/. We only used the ImageNet-1k training part and validation part for CATS Net training and testing in this work. Website: https://image-net.org/index.php. Website for CIFAR 100 dataset : https://www.cs.toronto.edu/~kriz/cifar.html

Research involving human participants, their data, or biological material

Policy information about studies with human participants or human data. See also policy information about sex, gender (identity/presentation), and sexual orientation and race, ethnicity and racism.

Reporting on sex and gender	Our findings apply to both sexes and genders. Sex and gender were not considered in our study design. We performed no sex- or gender-based analyses, because there was no sufficient evidence indicating differences in neural correlates of concept formation process between sexes or genders.
Reporting on race, ethnicity, or other socially relevant groupings	Our findings do not involve any racial or ethnic classfication.
Population characteristics	Participants were all right-handed and native Chinese speakers. None of them had experienced psychiatric or neurological disorders or had sustained a head injury. Twenty-nine participants (19 females; median age, 20 years; range, 18-32 years) were recruited in our study.
Recruitment	All participants were recruited online from colleage students in Beijing. Participant should be right-handed and nativeChinese speaker. None of them had experienced psychiatric or neurological disorders or had sustained a head injury. Eachparticipant read and signed the informed consent form before taking part in the experiments. Due to the college studentparticipants, the research results may not generalize to other populations (e.g., children).
Ethics oversight	All protocols and procedures of the current study were approved by the Ethics Committee of the State Key Laboratory of Cognitive Neuroscience and Learning at Beijing Normal University (ICBIR_A_0040_008).

Note that full information on the approval of the study protocol must also be provided in the manuscript.

Field-specific reporting

Please select the one below that is the best fit for your research. If you are not sure, read the appropriate sections before making your selection.

☐ Life sciences ☒ Behavioural & social sciences ☐ Ecological, evolutionary & environmental sciences

For a reference copy of the document with all sections, see nature.com/documents/nr-reporting-summary-flat.pdf

Behavioural & social sciences study design

All studies must disclose on these points even when the disclosure is negative.

Study description	This is a quantitative basic research involving human subjects.
Research sample	The sample sizes of the datasets was 29 (19 females; median age: 20 years; range: 18–32 years). The participants in this research are all adults, so they may not fully represent other groups (e.g., children).
Sampling strategy	None of the participants should have experienced psychiatric or neurological disorders or had sustained a head injury. All the participants should be all native Chinese Mandarin adult users in Beijing.<br>The sampling procedure was random designed. Sample sizes were determined by the previous model-fMRI alignment publications, and sample availability.
Data collection	In the fMRI experiments, participants' responses were recorded with a computer, while the ongoing brain activity during the task was recorded using a MRI scanner. The researcher was aware of the experimental conditions and the study hypothesis during data collection.

Timing	The fMRI dataset was collected in 2019-2020.
Data exclusions	The data of three participants were excluded from the analyses because of excessive head motion (> 3 mm/3°).
Non-participation	No participants declined participation or dropped out.
Randomization	Participants were not allocated into experimental groups.

Reporting for specific materials, systems and methods

We require information from authors about some types of materials, experimental systems and methods used in many studies. Here, indicate whether each material, system or method listed is relevant to your study. If you are not sure if a list item applies to your research, read the appropriate section before selecting a response.

Materials & experimental systems

n/a	Involved in the study
☒	☐ Antibodies
☒	☐ Eukaryotic cell lines
☒	☐ Palaeontology and archaeology
☒	☐ Animals and other organisms
☒	☐ Clinical data
☒	☐ Dual use research of concern
☒	☐ Plants

Methods

n/a	Involved in the study
☒	☐ ChIP-seq
☒	☐ Flow cytometry
☐	☒ MRI-based neuroimaging

Plants

Seed stocks	*Report on the source of all seed stocks or other plant material used. If applicable, state the seed stock centre and catalogue number. If plant specimens were collected from the field, describe the collection location, date and sampling procedures.*
Novel plant genotypes	*Describe the methods by which all novel plant genotypes were produced. This includes those generated by transgenic approaches, gene editing, chemical/radiation-based mutagenesis and hybridization. For transgenic lines, describe the transformation method, the number of independent lines analyzed and the generation upon which experiments were performed. For gene-edited lines, describe the editor used, the endogenous sequence targeted for editing, the targeting guide RNA sequence (if applicable) and how the editor was applied.*
Authentication	*Describe any authentication procedures for each seed stock used or novel genotype generated. Describe any experiments used to assess the effect of a mutation and, where applicable, how potential secondary effects (e.g. second site T-DNA insertions, mosiacism, off-target gene editing) were examined.*

Magnetic resonance imaging

Experimental design

Design type	Task-based fMRI (block design).
Design specifications	Ninety-five objects were chosen, including 3 common domains (32 animals, 35 small manipulable artefacts, and 28 large nonmanipulable artefacts). Each object was presented as a 400 × 400 pixels coloured image displaying a representative exemplar against a white background (10.55° × 10.55° of visual angle). All the participants were asked to name each displayed picture using oral language. The whole experiment included 6 runs, with each item repeated for 6 times across the experiment. Each run (8 min 45 s) consisted of 95 trials, with each item presented once per run. The trial structure consisted of a 0.5 s fixation, followed by a 0.8 s stimulus presentation and an intertrial interval (ITI) ranging from 2.7 s to 14.7 s.
Behavioral performance measures	There is no behaviour performance measures in this study.

Acquisition

Imaging type(s)	Functional, structural
Field strength	3 Tesla
Sequence & imaging parameters	Functional and anatomical MRI images were collected at the MRI center, Beijing Normal University using a 3 Tesla Siemens Trio Tim Scanner. A high-resolution 3D structural image was collected with a 3D magnetisation prepared-rapid gradient echo (3D-MPRAGE) sequence in the sagittal plane (144 slices, TR = 2530 ms, TE = 3.39 ms, flip angle = 7°, matrix size = 256 × 256, voxel size = 1.33 × 1 × 1.33 mm). Functional images were acquired with an echo-planar imaging

(EPI) sequence (33 axial slices, TR = 2000 ms, TE = 30 ms, flip angle = 90°, matrix size = 64 × 64, voxel size = 3 × 3 × 3.5 mm with a gap of 0.7 mm).

Area of acquisition: A whole brain scan.

Diffusion MRI: ☐ Used ☒ Not used

Preprocessing

Preprocessing software: The functional images were preprocessed and analyzed using Statistical Parametric Mapping (SPM12; http://www.fil.ion.ucl.ac.uk/spm).

Normalization: The images were normalized to Montreal Neurological Institute (MNI) space via unified segmentation (resampling into 3 × 3 × 3 mm^3 voxel size).

Normalization template: MNI305

Noise and artifact removal: For the preprocessing of the task fMRI data, the first five volumes of each functional run were discarded to reach signal equilibrium. Slice timing and 3-D head motion correction were performed. After normalization, the functional images were spatially smoothed using a 6-mm full-width-half-maximum Gaussian kernel for univariate analysis but not for multivariate pattern analysis. Temporal bandpass filtering (0.01–0.1 Hz) was performed to reduce the effects of high-frequency noises.

Volume censoring: None

Statistical modeling & inference

Model type and settings: Multivariate pattern analysis (MVPA); GLM analysis was first performed to obtain results of each regressors; during GLM analysis, six head motion parameters were included as nuisance regressors, and a high-pass filter (128 s) was used to remove low-frequency signal drift for each run; then MVPA were performed; the results of MVPA were entered into second-level (between-subject) random-effect analysis.

Effect(s) tested: Representational similarity analyses; partial Spearman's correlation.

Specify type of analysis: ☐ Whole brain ☐ ROI-based ☒ Both

Anatomical location(s): The VOTC mask was defined as regions showing stronger activation to all pictures relative to the rest in the fMRI dataset (FDR q < 0.05) within the cerebral mask combining the posterior and temporooccipital divisions of inferior temporal gyrus (15#, 16#), the inferior division of lateral occipital cortex (23#), the posterior division of parahippocampal gyrus (35#), the lingual gyrus (36#), the posterior division of temporal fusiform cortex (38#), the temporal occipital fusiform cortex (39#), the occipital fusiform gyrus (40#), the supracalcarine cortex (47#) and the occipital pole (48#) in the Harvard-Oxford Atlas (probability > 0.2).

Statistic type for inference (See Eklund et al. 2016): All effects in whole-brain level were tested by one sample t-tests and cluster-wise FWE correction as implemented in SPM12. Effects at ROI level were tested by null-hypothesis one, pair and independent sample ttests.

Correction: For whole-brain analysis, multiple comparison corrections were conducted using cluster-level FWE correction (p <.05) asimplemented in SPM12 (voxel-wise p <.001).

Models & analysis

n/a	Involved in the study
☒	☐ Functional and/or effective connectivity
☒	☐ Graph analysis
☐	☒ Multivariate modeling or predictive analysis

Multivariate modeling and predictive analysis: We conducted searchlight MVPA within a ventral occipitotemporal cortex (VOTC) mask. This mask was defined on the basis of regions showing stronger activation to all pictures relative to baseline in hearing participants from the fmri dataset (q < 0.05, FDR-corrected). The functional mask was further constrained by the following anatomical parcels (Harvard–Oxford Atlas, probability > 0.2): the posterior and temporooccipital divisions of the inferior temporal gyrus (15#, 16#), the inferior division of the lateral occipital cortex (23#), the posterior division of the parahippocampal gyrus (35#), the lingual gyrus (36#), the posterior division of the temporal fusiform cortex (38#), the temporal occipital fusiform cortex (39#), the occipital fusiform gyrus (40#), the supracalcarine cortex (47#), and the occipital pole (48#). This definition resulted in the selection of 2467 voxels in the left hemisphere and 2420 voxels in the right hemisphere.

For each voxel within the VOTC mask, multivariate activation patterns within a sphere (radius = 10 mm) centred at that voxel were extracted. Neural RDMs were computed with the Pearson distance within the searchlight sphere. Then, Spearman's rank correlation coefficients between the neural RDM and model-derived concept RDMs was computed, controlling for the effects of sensory input layer. Correlation maps were obtained for each participant by moving the searchlight centre across the VOTC mask. These maps

were Fisher z-transformed and spatially smoothed with a 6 mm full-width half-maximum (FWHM) Gaussian kernel. The correlation maps were compared to 0 with one-tailed one-sample t tests.

ROI-based MVPA was conducted with the same VOTC mask, semantic control mask and multiple demand network mask as ROIs. Specifically, multivariate activity patterns for each stimulus within the ROI mask were extracted. Neural RDMs were computed on the basis of Pearson distances and then correlated with the RDM generated by conceptl layer and CA layers. The resulting correlation coefficients between the neural and model RDMs were Fisher z-transformed and compared to zero with one-tailed one-sample t tests.
